# Building spatial composite indicators to analyze environmental health inequalities on a regional scale

**DOI:** 10.1186/s12940-015-0054-3

**Published:** 2015-08-21

**Authors:** Mahdi-Salim Saib, Julien Caudeville, Maxime Beauchamp, Florence Carré, Olivier Ganry, Alain Trugeon, Andre Cicolella

**Affiliations:** French National Institute for Industrial Environment and Risks, Parc Technologique Alata,BP 2, 60550 Verneuil-en-Halatte, France; Epidemiology and public health department, University hospital of Amiens, Amiens, France; Regional Observatory of Health and Social Issues in Picardie, Amiens, France

**Keywords:** Principal Component Analysis, Spatial, Heterogeneity, Autocorrelation, Deprivation, Exposure, Health, Composite indicators

## Abstract

**Background:**

Reducing health inequalities involves the identification and characterization of social and exposure factors and the way they accumulate in a given area. The areas of accumulation then allow for prioritization of interventions. The present study aims to build spatial composite indicators based on the aggregation of environmental, social and health indicators and their inter-relationships.

**Method:**

Preliminary work was carried out firstly to homogenize spatial coverage, and secondly to study spatial variation of environmental (EI), socioeconomic (SI) and health (HI) indicators. The aggregation of the different indicators was performed using several methodologies for which results and decision-makers’ usability were compared.

**Results:**

Four methodologies were tested: 1) A simple summation of normalized HI, EI and SI indicators (IC), 2) the sum of the normalized HI, EI and SI indicators weighted by the first principal component of a Principal Component Analysis (IC PCA), 3) the sum of normalized and weighted indicators of the first principal component of Local Principal Component Analysis (IC LPCA), and 4) the sum of normalized and weighted indicators of the first principal component of a Geographically Weighted Principal Component Analysis (IC GWPCA).

**Conclusion:**

The GWPCA is particularly adapted to taking into account the spatial heterogeneity and the spatial autocorrelation between SI, EI and HI. This approach invalidates the basic assumptions of many standard statistical analyses. Where socioeconomic indicators present high deprivation and where they are associated with potential modifiable health determinants, decision-makers can prioritize these areas for reducing inequalities by controlling the socioeconomic and health determinants.

## Background

Analyzing the relationship between environment and health has become a major issue for public health as stressed in the second French National Plan for Health and Environment (NPHE). In 2004, French ministries involved in the study of the impact of the environment on health published the NPHE describing what action the government intends to take in this field over a period of five years. Two priority areas were selected: preventing health risks related to the quality of resources on the one hand and to chemicals on the other; and developing environmental health through research, expertise, training and information. The second NPHE, a successor of the first plan, was prepared in view of the next conference “Health and Environment” organized by the World Health Organization.

Two main axes were adopted:

- to identify and manage geographic areas where hotspot exposure is suspected of generating a potential hazard to human health owing to exposure at stances, which may be present in air, soil, water, foods, as consequence of anthropic activities.
- to reduce exposure inequalities.

Geographic inequality has become a primordial topic that comes to guide policy development in France [1]. The recent WHO report on Environmental health on inequalities in Europe indicates that the lower socioeconomic groups are often both potentially overexposed to environmental pollution and vulnerable to the health effects resulting from this pollution [2]. As a result, reducing health inequalities involves the identification and characterization of social and exposure factors in order to interpret how they accumulate in an area in order to identify and prioritize interventions. The health status of a given population is the result of complex interactions between many social, territorial and environmental factors. Their associated combinations with individual determinants influence the health of a population.

The robustness and the reliability of the cross-analysis approaches in terms of management raises a number of questions, not least concerning the evaluation of the phenomena resulting from the difficulty to assess processes as evidenced by the emergence of the concept of the exposome [3] and, secondly, in view of the representativeness of available variables and the specificity of statistical analysis considering spatial data. The development of a method that enables a characterization of the accumulation of Territorial, Environmental and Social Health Inequalities (TESHI) is an essential prerequisite to the implementation of public health politics aimed at protecting the population.

A share-of-population census, monitoring of environmental quality and health data collection were conducted independently of each other according to specific needs and constraints. These data have already made it possible to highlight important regional disparities both in terms of the distribution of disease [4–6] as well as on the environmental quality [7–9]. Data are often available at a fine administrative level or resolution and enable building of environmental, socioeconomic and health indicators on a regional scale. The definition of indicators for the identification and, characterization of environmental and social health inequalities depends on the reutilization of this type of data, which is very diverse by nature with regard to its initial intended objectives [10]. The construction of composite indicators is needed to provide diagnostics at a territorial level by integrating various environmental, socioeconomic and health dimensions. The definition of a cumulative indicator that combines different dimensions is broad and does not suggest a specific process. Different approaches have been already tested to combine variables [11, 12]. Some indicators are compiled using randomly weighted variables or according to certain objectives [13, 14]. For example, a regional socioeconomic index (BC Stats) has been developed simply by combining six variables which had an uneven weight in the final calculation of the index [13]. Other composite indicators are based on an equal weighting between all variables, resulting in an additive aggregation of variables – as it was the case with the combination of air pollution indicators and social vulnerability indicators [11]. Several other synthetic indices use PCA for determining weights, such as the “Index of Multiple Deprivation (UK)” created in 2004 and updated in 2007 (Index of Multiple Deprivation 2007 or IMD 2007) and 2010 (IMD 2010). This index is constructed from 37 variables divided into seven areas: income, employment, health, education, access / barriers to services, residential environment, and crime [15]. The PCA method permits to take variable collinearity into account, thereby avoiding redundant information. However, these approaches do not integrate the specificities linked to the handling of spatial data processing, positional and/or attribute information. One of the main features of these data is spatial autocorrelation, which measures the degree of interaction and interdependence between spatially located observations [16].

Another one is spatial heterogeneity, which refers to the non-stationary nature of geographical processes. This means that processes vary locally and are not necessarily the same at each position in geographic space [16]. These spatial phenomena invalidate one of the basic statistical assumptions, which is that data are independent and identically distributed in space. In the environmental health field, spatial statistics have addressed this issue, and the most commonly used methods are the geographically weighted models (GW) developed by Fotheringham et al. [17–19]. These models have recently been identified as geostatistical methods that should be encouraged in health studies [20]. In particular, Harris [21] recently implemented the geographically weighted PCA (GWPCA) to replace the standard PCA. GWPCA is adapted to account for spatial autocorrelation and heterogeneities in the spatial process. However, these techniques had never been applied to building composite Health-Environment indicators.

The present study aims to characterize and integrate spatial phenomena represented by spatial indicators and to combine them to create a composite indicator useful for evaluating areas where environmental and social health inequalities are accumulating. Different methodologies are for building the composite indicators, some integrating spatial processes, while others do not. The results and utility for potential decision-makers are also evaluated.

## Materials and methods

### Data

**Fig. 1:**
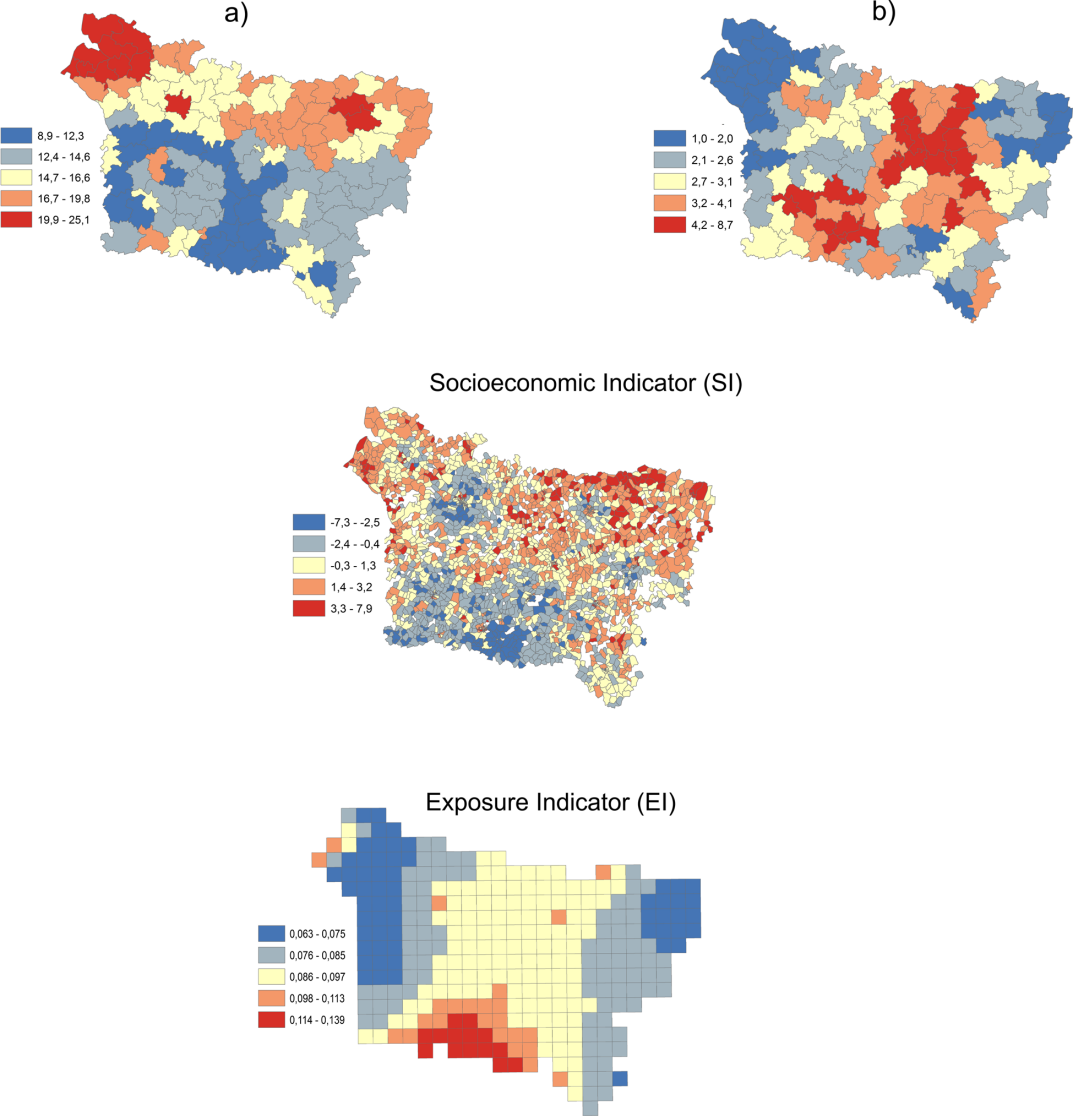
Health indicator (HI). **a** Lip. Oral Cavity and Pharynx Canter Mortality and **b** Pleural cancer mortality

The indicators used to characterize the three dimensions (social, health and environmental) (Fig.1) have been defined and analyzed in a previous study in order to quantify the spatial relationship between these indicators [22]. They are:

#### Inhalation Exposure Indicators (EI)

The environmental inhalation exposure indicators used were those described in Caudeville et al. for building a GIS-based modeling platform for quantifying human exposure to chemical stances (PLAINE: environmental inequalities analysis platform) [23]. The exposure indicators integrate atmospheric concentration data to construct population exposure indicators at a fine resolution (10 x 15 km grid) based on the modeling of trace metals (nickel-Ni, cadmium-Cd, and lead-Pb) transportation within the Picardy region [24].

#### Socioeconomic Indicator (SI)

The deprivation indicator (FDep) used was developed by Rey [25]. The concept of the urban unit developed by the National Institute of Statistics and Economic Studies (INSEE) was used to define the degree of urbanicity. There are five categories of urban unit: rural (less than 2,000 people), quasi-rural (population 2000 to 9999), quasi-urban (population of 10 000–99 999), urban (population of 100,000 to 1,999,999) and-suburban (population >2,000,000). The indicator was built at the French census block level (called IRIS “Îlot Regroupé pour l’Information Statistique”) IRIS using the following socioeconomic variables: median household income, percentage of high school graduates in the population aged 15 and over, the percentage blue-collar workers in the active population and the unemployment rate. The socio-economic index (SI) was defined as the weighted sum of these four variables by the first principal component of PCA and stratified in four degrees of district classes of urbanicity.

#### Health Indicator (HI)

Individual-level mortality records (including age, sex, cause of death and area of residence at death) were obtained from the Inserm-CépiDc database for the mainland Picardy region. The age-adjusted cancer mortality rates are calculated for each county from 2000 to 2009 by the Regional Health Observatory of Picardy [26] and smoothed using a geostatistical method (Poisson kriging) to incorporate the size and shape of administrative units as well as the population density into the filtering of noisy mortality rates [22].

### Spatial scale

Analyses of correlations between health data and putative factors are traditionally performed using a global or “aspatial” regression model, under the implicit assumption that the impact of variables is constant over the entire study area. This assumption is likely unrealistic for large areas, which can display large geographic variations. Fotheringham and colleagues developed Geographically Weighted Regression (GWR) to explore spatial non-stationarity and map statistics to visualize the spatial patterns of the relationships between dependent and independent variables [17, 18].

The GWR has previously been used to quantify the relationship between these dimensions with the Health Indicator (HI) as the dependent variable and Socioeconomic Indicator (SI) and exposure indicator ETM (by ingestion and inhalation) as independent variables [22]. The results strongly suggest that the relationships between cancer (lip, oral cavity and pharynx-pleural) mortality and the environmental and deprivation indexes are not stationary but instead vary over the study area. This exploratory analysis also allowed us to assess the choice of the resolution of the spatial analysis; two scales were used:

- The county level, because pleural cancer (HI = pleural) is correlated with EI, and pleural cancer and EI are characterized by positive spatial autocorrelation at the IRIS and county levels.
- The IRIS level, because lip cancer (HI = lip) is correlated with SI, and SI is affected by the use of different spatial structures (the variance and spatial autocorrelation decreased with increasing aggregation size).

In this previous study, we used a bi-square kernel and the distance function, which is characterized by a bandwidth that corresponds to the distance beyond which the weight rapidly approaches zero. The local regression was based on the following number of closest observations, which represented 15 %–20 % of the data: 22 for the county level, and 426 for the IRIS level. The following bandwidths were found to be optimal: lip, oral cavity and pharynx cancer mortality (47 km), and pleural cancer mortality (54 km).

Table 1 shows spatially resolved data types and approaches used to homogenize spatial coverage.

**Table 1:** Spatially resolved data types and approaches used to homogenize spatial coverage

Indicator	Variables	Sources	Resolution and variable combination	Spatial operation
Socioeconomic Indicator (SI)	Median household income	French census	Vector data from the IRIS.	Spatial population-weighted aggregation
	Percentage high school graduates	Rey *et al*. [25]		
	Percentage workers			
	Unemployment rate			
Exposure Indicator (EI)	- Nickel-Ni,	Caudeville *et al*. [24].	Raster data of 1 km^2^ grid	Spatial aggregation
	- Cadmium-Cd,			
	- Lead-Pb			
Health Indicator (HI)	Lip, oral cavity and pharynx cancer mortality	Regional Health Observatory of	Vector data from the county database	Poisson kriging
	Pleural cancer mortality			
		Picardy. [22].		

### Methods

Four methods for combining indicators were explored in the present paper; 1) A simple summation of normalized HI, EI and SI indicators (IC), 2) the first principal component analysis of the normalized HI, EI and SI indicators (IC PCA) to account for correlation between indicators (IC PCA), 3) the first principal component of LPCA of the normalized HI, EI and SI indicators to account for spatial autocorrelation and correlation between indicators (IC LPCA), and 4) the first principal component of GWPCA of the normalized HI, EI and SI indicators to account both for spatial heterogeneity and spatial autocorrelation (IC GWPCA). The latter three methods enable redundancy of information between the three indicators to be avoided. This paper does not give the full detail of those procedures that have been already published:

#### Principal components

The former is a classical PCA analysis and is one of the most popular dimensionality reduction methods. It is a linear method, meaning that the transformation between the original data and the new lower dimensional representation is a linear projection. Its main purpose is dimensionality reduction, but it can also be used to explore relationships between variables. This methods has been fully described by Jolliffe et al. [27].

#### Local principal components

The aim of the LPCA is to take into account the spatial autocorrelation. This information is introduced by the way of a spatial weighting matrix $C=\left[{\displaystyle {c}_{{}_{ij}}}\right]$ that indicates the strength of the relationship between units *i* and *j*. This matrix can take many forms, for example a binary connectivity matrix B (*b*_*ij*_ = 1 if units *i* and *j* are neighbours, else *b*_*ij*_ = 0). Here, This matrix B is transformed into tied distances , where d is the size of the set of *S*, the group of points that are equidistant from ego that contains the *k*^*th*^ nearest neighbor, and a is the number of points before the first member of *S*. So the group of weights that would be included in calculations for ego is *w*_0_, *w*_1_, …, *w*_*a*_, *w*_*a* + 1_, …, *w*_*k*_, …, *w*_*a* + *d*_, where *w*_*i*_ = *1* for *i* values from *0* to *a*, and *w*_*i*_ = *(k-a)/d* for values of *w* from *a+1* to *a+d*. In the case of a correlation matrix PCA, the LPCA analysis is equivalent to Wartenberg's Multi-variate Spatial Correlation Analysis [28]. See Dray et al [29] for more details about these approach.

#### Geographically weighted principal components

The latter techniques adopt a nonparametric, kernel-based approach and is termed geographically weighted PCA (GWPCA). The GWPCA technique can be viewed as a direct alternative to SPCA for incorporating spatial effects into a PCA, but whereas GWPCA accounts for first order (nonstationary) spatial effects, sPCA accounts for second-order (stationary) spatial effects. Such methodological differences are analogous to the use of a GWR (The weighting is controlled by a weighting function) or a regression with a spatially auto correlated (error term) when choosing a regression model to study spatially referenced data. In this case study, GWPCA is calibrated with bandwidth supplied exogenously, already estimated for GWR in the previous study [22]. See Harris et al. [21] for more details about these approach.

## Results

Tables 2 and 3 show the correlation between HI, SI, and EI. HI = lip is significantly and positively correlated with the SI and significantly and negatively correlated with EI (Table 2), and HI = pleural is significantly and positively correlated with EI and negatively correlated with SI (Table 3), respectively. This statistic also shows that SI is significantly and negatively correlated with EI for both examples. It is noteworthy that the correlation coefficients were higher at the county level than at the IRIS level, which was the expected result because aggregation is known to increase the strength of correlation [30].

**Table 2:** Pearson’s Correlation Matrix of HI = lip, SI and EI at IRIS level

Variables	Health Indicator HI)	Exposure Indicator (EI)	Socioeconomic Indicator (SI)
Health Indicator (HI)	1.0*	−0.20	0.36*
Exposure Indicator (EI)		1.0*	−0.28*
Socioeconomic Indicator(SI)			1.0*

*Significant p < 0.01

**Table 3:** Pearson’s Correlation Matrix of HI = pleura, SI and EI at county level

Variables	Health Indicator (HI)	Exposure Indicator (EI)	Socioeconomic Indicator (SI)
Health Indicator (HI)	1.0*	0.51*	−0.18
Exposure Indicator (EI)		1.0*	−0.50*
Socioeconomic Indicator (SI)			1.0*

*Significant p < 0.01

The global PCA reveals that the first components have eigenvalues greater than or very close to unity and that they account for 62 % at the county level (HI = pleura) (Table 4) and 52 % at the IRIS level (HI = lip) (Table 5) of the variation in the data. The LPCA reveals also that the first principal component has greater eigenvalues and that they account for 82 % (HI = pleura) and 79 % (HI = lip) of the variation in the data. The percentage of variance explained by the first principal component is substantially higher with LPCA than with global PCA for the two examples.

**Table 4:** Estimated weights coefficients using the first principal component of *PCA* and *LW PCA* for each indicators at the county level when HI = pleural cancer mortality

Variables	PCA	LPCA (54 km)
Health Indicator (HI)	0.53	0.68
Exposure Indicator (EI)	0.66	0.81
Socioeconomic Indicator (SI)	−0.56	−0.73
% variable	0.62	0.82
Eigenvalues	1.88	1.50

**Table 5:** The weights coefficients estimated by the first principal component of *PCA* and *LW PCA* for each indicators at the IRIS level when Hi = lip. Oral cavity and pharynx cancer mortality

Variables	PCA	LPCA (47 km)
Health Indicator (HI)	0.58	0.76
Exposure Indicator (EI)	−0.51	−0.71
Socioeconomic Indicator (SI)	0.62	0.66
% variable	0.52	0.79
Eigenvalues	1.57	1.86

Taking the information provided by neighboring units into account improves the percentage of variance explained by the first component. For both types of geographical units, and for both examples, the global PCA and LPCA show that the three indicators are strongly correlated with the first component: negatively with SI, and positively with HI and EI, for example, when HI = pleura (Table 4), and negatively with EI, and positively with HI and SI, for example, when HI = lip (Table 5). Note that global PCA, as with any global summary, captures general trends but may mask marked local variation effects, which are often vital to a more complete understanding of a given process.

Tables 6 and 7 show the correlation between IC, IC PCA, IC LPCA and IC GWPCA for HI = lip and pleura. We found a Pearson’s correlation equal to 0.52 and 0.55 between IC and IC PCA, respectively, for HI = lip and HI = pleura. The strongest correlation coefficients are found between standard and local weight PCA (0.88 and 0.90).

**Table 6:** Pearson’s correlation matrix of IC, ICPCA, LPCA and GWPCA when HI = lip

Indicators	IC	IC PCA	LPCA	GWPCA
IC	1.0	0.52	0.43	0.53
IC PCA		1.0	0.98	0.82
IC LPCA			1.0	0.80
IC GWPCA				1.0

**Table 7:** Pearson’s Correlation Matrix of IC, ICPCA, LPCA and GWPCA when HI = pleura

Indicators	IC	IC PCA	LPCA	GWPCA
IC	1.0	0.55	0.52	0.65
IC PCA		1.0	0.99	0.67
IC LPCA			1.0	0.65
IC GWPCA				1.0

**Fig. 2:**
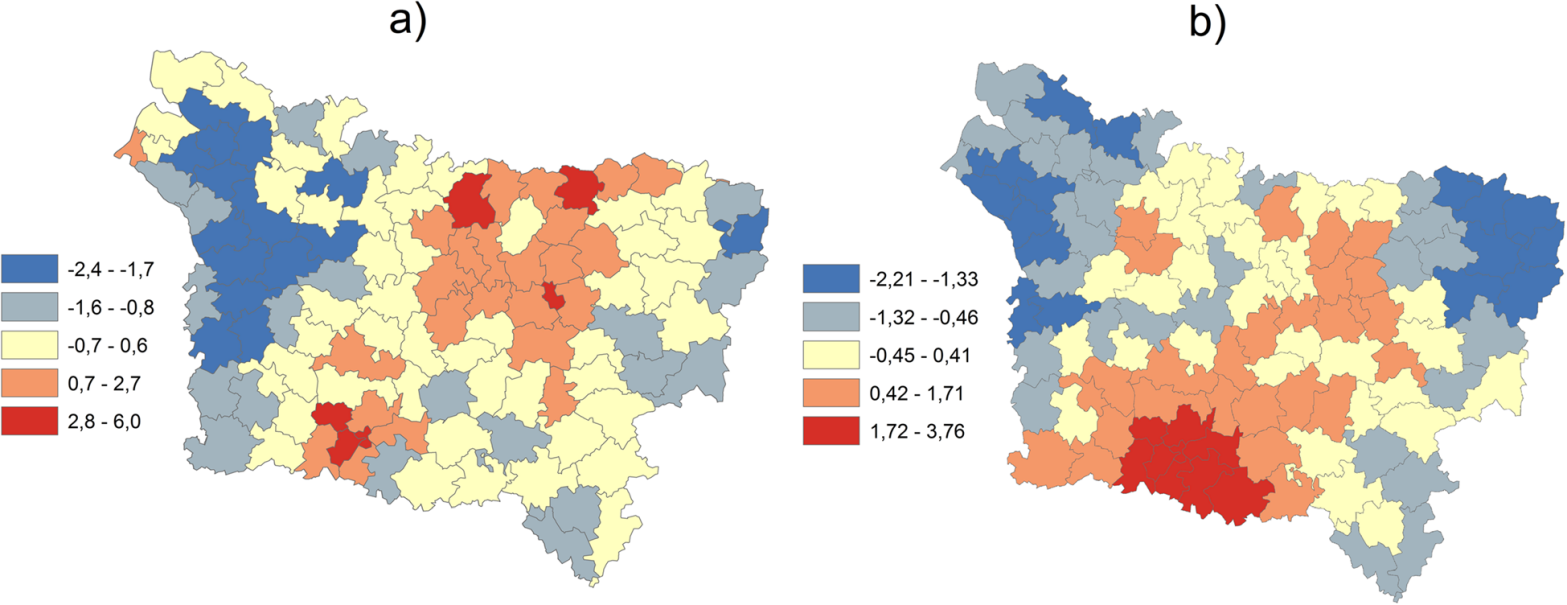
The composite indicator when HI = pleura represented by: **a** the summation of normalized indicators (IC) **b** the summation of normalized and weighted indicators by the first component of PCA (IC PCA)

Figure 2a shows the composite indicator represented by the summation of normalized HI, EI and SI indicators (IC). An area with a high score would be expected to experience much higher levels of deprivation than areas with low scores. Note that summation means equal weighting, and in any case, equal weighting does not mean “no weight,” but implicitly implies, that the weights are equal.

Figure 2b shows the composite indicator represented by the summation of normalized indicators weighted by the first principal component of PCA. Due to the fact that these indicators are correlated (Table 2), an equal weighting (the summation) may introduce an element of double counting into the IC indicator. The spatial pattern differences between the IC and IC PCA maps (Fig. 2) are due to correlation integration in the PCA method and explain the low correlation coefficient between IC and IC PCA (Tables 6 and 7). Take note that the PCA method implicitly assumed that the correlation between indicators is constant across the study area. This assumption is likely unrealistic for large areas, which can display substantial geographic variation in socioeconomic and environmental conditions and is corrected by using the GW PCA method.

**Fig. 3:**
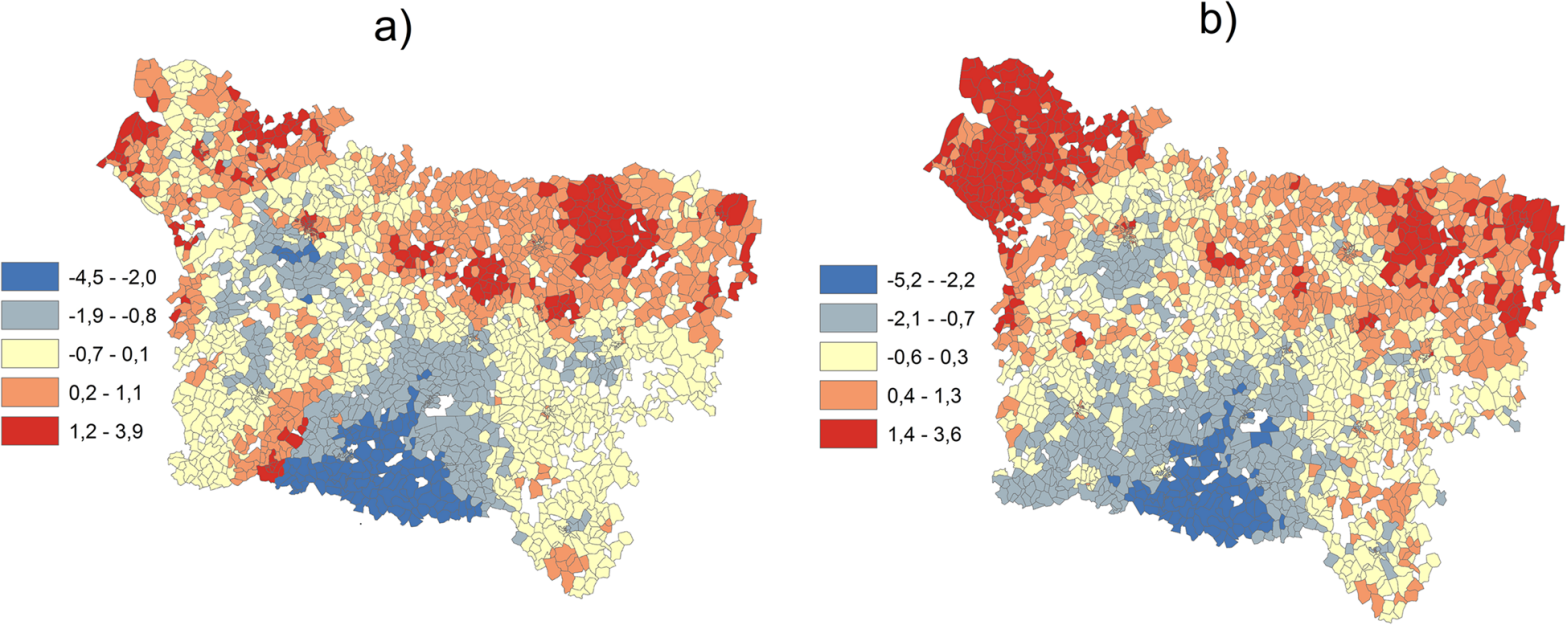
The composite indicator when HI = lip represented by **a** the summation of normalized and weighted indicators by the first principal component of GWPCA (IC GWPCA), **b** the summation of normalized and weighted indicators by the first principal component of PCA (IC PCA)

**Fig. 4:**
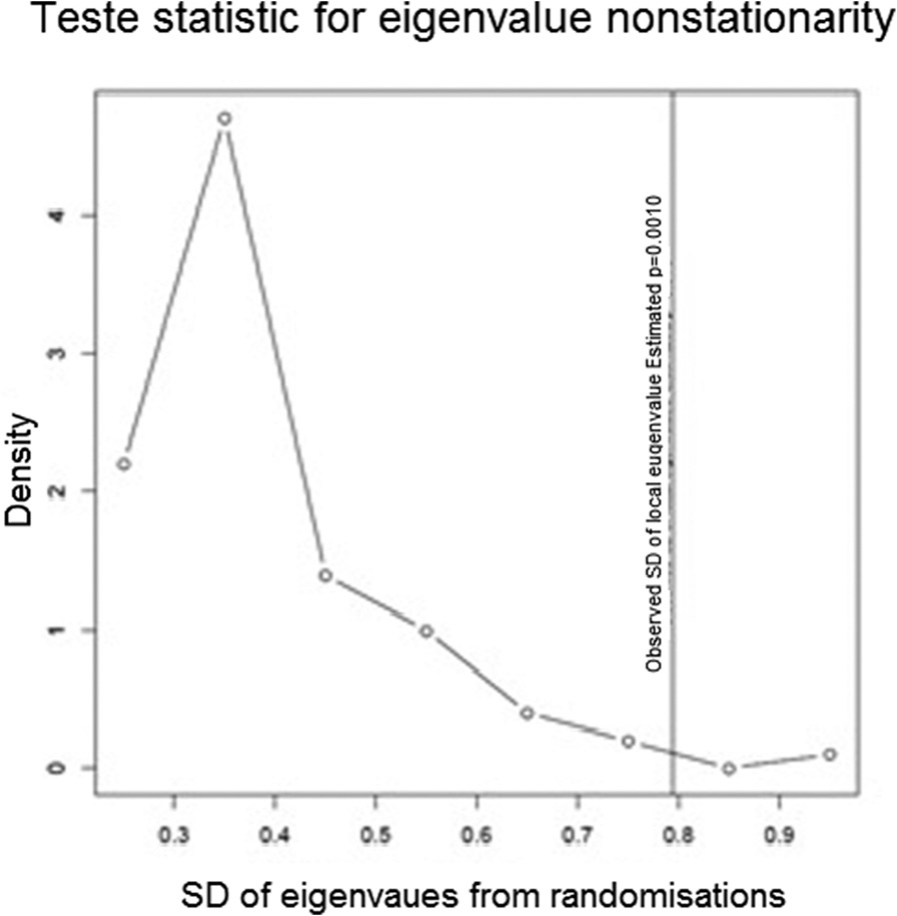
Randomization test for eigenvalue for HI = pleura

Figure 3a shows the composite indicator represented by the summation of normalized indicators weighted by the first principal component of GWPCA. The maps show a spatial structure slightly different from this presented by the map of IC PCA (3b). This is due to the fact that the correlation between the three indicators is non-stationary and varies in the study area, as shows the Monte Carlo tests used to evaluate whether local eigenvalues from GWPCA vary significantly across space (Fig. 4). As the p-value for the true SD is very small (p =0.010), the null hypothesis of local eigenvalue non-stationarity is rejected (see Harris et al. 2011 for more details for this test).

**Fig. 5:**
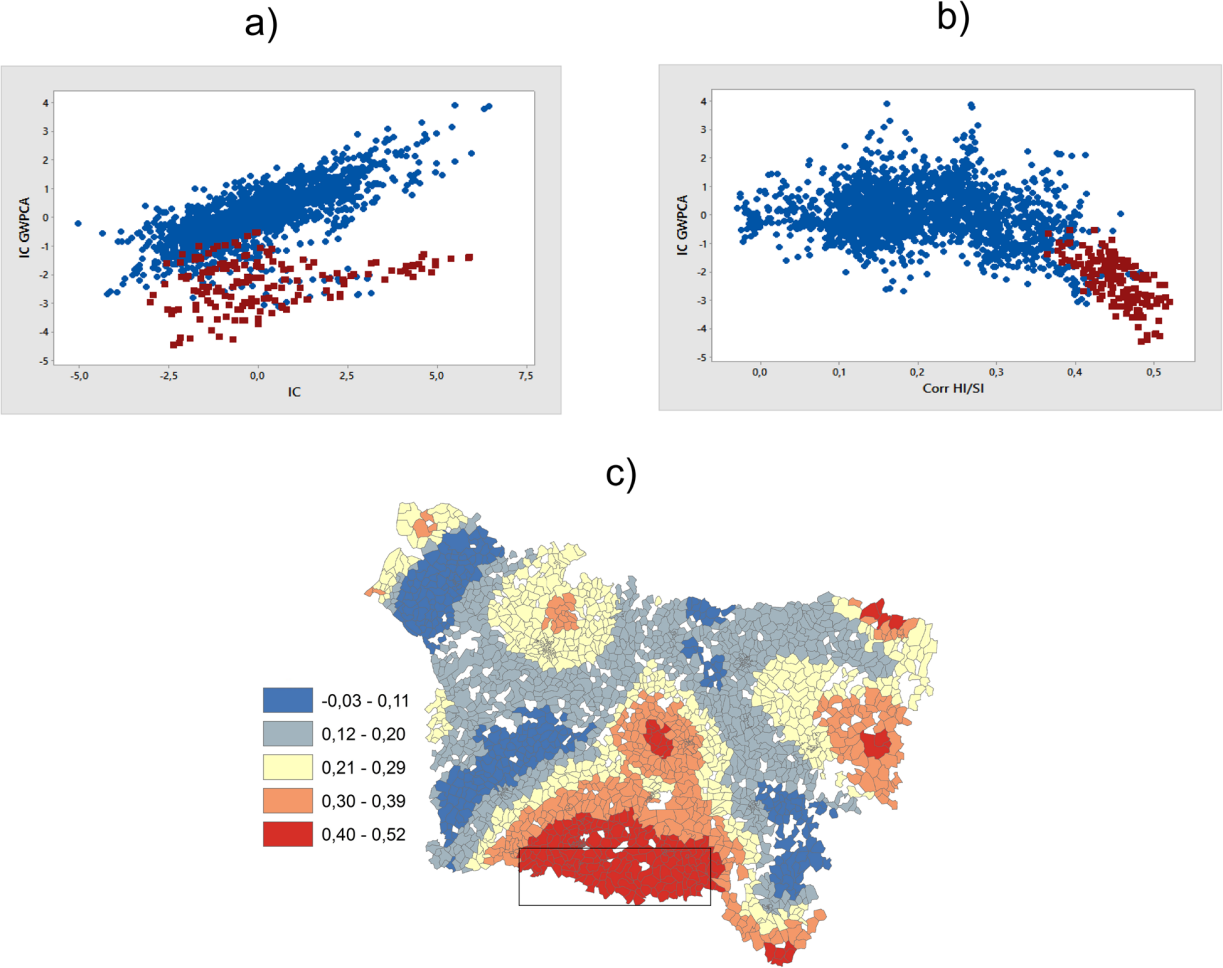
The scatter plots **a** between IC GWPCA and IC **b** IC GWPCA and local correlation coefficient HI and SI, and **c** map of the local correlation coefficient between HI = lip and SI estimated by GWR (see Saib et al. [22])

Figure 5 shows the scatter plots a) between IC GWPCA and IC b) IC GWPCA and local correlation coefficient HI and SI, and c) Map of the local correlation coefficient between HI = lip and SI estimated by GWR [22].

The scatterplot (a) shows that some areas with a low IC GWPCA are associated with an elevated IC. These areas correspond to IRIS with strong local correlation coefficient between HI and SI identified by GWR (Fig. 5c). These results show the local correlation variation integration effect on the calculation of the composite indicators using the GWPCA method.

## Discussion

Over the last few decades, there has been an increase in the number of composite indicators developed by various national and international agencies. Unfortunately, individual indicators are sometimes selected in an arbitrary manner with little attention paid to the interrelationships between them. This can lead to indices which overwhelm, confuse and mislead decision-makers and the general public.

The underlying nature of the data needs to be analyzed carefully before a composite indicator is constructed. This preliminary step is helpful in assessing the suitability of the data set and will provide an understanding of the implications of the methodological choices depending on the interrelationships between them. The set of indicators used in this study to characterize the three social, health and environmental dimensions in the Picardy region have already been used in a previous study. The aim was to quantify the relationship between these dimensions.

In this study, the results of geographically weighted regression with the health indicator as the dependent variable showed the existence of spatial variations in the structure of the relationship between these dimensions. We have evaluated the impact of the MAUP effect and defined spatial heterogeneity of potential cofactor effect on the analysis. It is feasible that our environmental indicator lacks the spatial representativeness to fully capture the environmental effects on health that we have evaluated. Pollution levels vary relatively on the local scale; making precise estimates of exposure on opposite spatial scales is a primary consideration when evaluating the relationship between air pollution and health [31, 32].

The results of this study and the comparison of different approaches used to build accumulation indicators has enabled us to better understand and interpret the accumulation and interrelation of these inequalities in a given territory and to partially overcome problems of using spatial aggregated data.

PCA is a widely-used means of reducing dimensionality and identifying combinations of characteristics in many different disciplines (see Jolliffe, [27]). Although the comparison between the IC indicator and IC PCA (Fig. 2) allows us to have general ideas about the accumulation of these inequality (social environmental and health), and although], however in the case of spatial data, such as the -indicators used in this study, global PCA ignores any spatial characteristics in the data. Nonetheless, such effects are often critical to a more complete understanding of a given process [21]. For that reason, the analysis has been expanded to take into account two properties of spatial data, spatial heterogeneity and spatial autocorrelation, which invalidates the basic assumptions of many standard statistical analyses: that data are independently generated and identically distributed [33].

The three indicators used in this study are characterized by a spatial autocorrelation [22] and the percentage of explained variance is substantially higher with LACP than with global PCA (Tables 4 and 5). The integration of the spatial autocorrelation in the analysis allows a better description of the phenomenon. Indeed, LPCA is applied to the situation when the data are not described well by a universal set of PCA but where there are localized regions in attribute data space where a suitably localized set of PCs provide a better description [34–37].

The results of the application of GWR in a previous study [22] suggest that the relationships between indicators are not stationary but instead vary over the study area. The application of the GWPCA allows to take this non-stationarity of relations between the three indicators into account (social, health and environmental); for example, Fig. 4 shows that the lowest score of the GWPCA in comparison with the IC is located in south of the region (yellow points in the figure). This corresponds to areas with a strong local correlation coefficient between health and socioeconomic indicators. The GWPCA has obvious benefits in that it can account for local differences in the spatial scale of variation of the variables utilized. Note that one problem some cite as a drawback to the method is obtaining a clear pattern of loadings, which is why we conducted rotation in for some units for results of GWPCA (the sum of eigenvalues is not affected by rotation, but changing the axes will alter the eigenvalues of particular factors) [38].

In this study, the indicators were chosen because they were built to define the dimensions to be considered (social, health and environmental) based on the realities to be measured, availability and quality of data. For example, the exposure indicator (EI) was set up by an approach that takes the principal exposure pathways to integrating local and global pollutant sources from the past and present [24] and the major routes of exposure into account. Several socioeconomic indicators were built in France. To approach social situations on the basis of geo-referenced information, we selected the FDep indicator due to the properties it offers: it is one-dimensional, maximizing the representation of the heterogeneity of its components and strongly associated with the components stratified in different urban criteria to better integrate the rural/urban gradient [25]. The health indicator smoothed using a geostatistical method for filtering noise caused by the “small number problem” allowed also us to estimate mortality risk on different spatial scales, which facilitated the analysis of the relationships of cancer mortality rates with environmental and socioeconomic data measured on very different scales [22].

However, this interpretation should be treated with caution. Our study has limitations that highlight some future research priorities. We recognize that the environmental indicators used in the study constitute only a few pieces of existing global environmental inequalities: there are other environmental characteristics that are important for health that is not possible to take in account in that kind of indicator due to data availability. For the same reason, there are also confounding factors at the individual level that have not been accounted for, such as smoking, alcohol consumption and physical activity. We have assumed that residential location is an adequate proxy for environmental exposure, but migration and movement may make the locations of human exposure likely to be not exactly the same.

Our study therefore cannot ascertain causality, and consequently it is not possible to establish whether the correlation estimated constitutes a real risk factor and a biological plausibility to epidemiological observations. For this specific study, incidence data should be more suitable than mortality; indeed the development of diseases often requires a long-term human exposure to environmental risk. It would be useful for future research to consider the temporal course of environmental risk exposure and health. While we examined the spatial relationship between environmental and socioeconomic dimensions and specific cause, our analyses did not consider adapted indicators. For a stakeholder in need of information to guide their actions to reduce these inequalities, the choice of indicator has to be adapted to the local context and action options. Therefore, an examination of health outcomes that have an established causal link with the environmental or social aspect may have led to defining other relevant indicators.

Although the use of these different approaches to construct a composite indicator for the accumulation of the Territorial, Environmental and Social Health Inequalities has some advantages, such as avoiding redundancy of information and the inclusion of spatial characteristics for data, they are not fully suitable for a stakeholder who is in need of information to guide their actions in order to reduce these inequalities.

From a stakeholder’s point of view, addressing the numerous and multifaceted inequalities is more potent when it characterizes the social and environmental processes that determine unequal health indicator distribution in order to point out potential health determinants. Those approaches suggest that the effectiveness of deprivation maps for making decisions for safeguarding citizen health involves building a supplementary map capable of highlighting areas where health indicators with high values are correlated with environment and/or socioeconomic factors. This additional information should correspond to the difference between the simple cumulative and the integrated spatial process indicator results in order to characterize local relationships. Associated with the simple cumulative indicator, this map makes it possible to highlight potential determinants on the basis of which stakeholders could act and guide health policy in areas where deprivation is strong and determinants might potentially be changed.

## Conclusions

In conclusion, this study proposes a methodology for combining environmental, socioeconomic and health indicators in the context of the French NEHP by using a suite of statewide indicators to characterize both pollution burden and population characteristics. It uses a scoring system to weight and sum each set of indicators within pollution burden and population characteristics components by taking data spatially processed at the local, intermediate and regional scale in account.

The spatial relationship characterization of these three indicators could be performed by comparing the different method results. This additional spatial information has important implications for policymakers and could be used to prioritize actions in areas where deprivation is elevated and associated with potentially modifiable health determinants.

In particular, there is a pressing need to use individual-level quantitative analysis to evaluate how vulnerability among different social and demographic groups interacts with multiple environmental deprivations to determine the genesis and perpetuation of vulnerability.

The results of the different approaches used to build the accumulation indicator has allowed for a better understanding and interpretation of the process of accumulation of these inequalities in a given territory and to partially overcome the problems associated with using aggregated data. By integrating the produced result into the PLAINE platform, it will be possible to create a “source-effect chain” through intermediate paths (pollution sources, biomonitoring measurements, etc.). On a regional scale and for a fine resolution of exposure outcome prerequisites, environmental monitoring networks are not sufficient to characterize the multidimensionality of the exposure concept. In an attempt to increase representativeness of spatial exposure assessment approaches, this kind of methodology could be developed using additional available databases and theoretical framework approaches to combine factor risks and exposure at different conceptual levels of characterization.
